# Richmond Academy of Medicine and Surgery

**Published:** 1897-01

**Authors:** John N. Upshur

**Affiliations:** Richmond, Va., Professor Practice of Medicine in the Medical College of Virginia, Etc.


### Society Notes.

### RICHMOND ACADEMY OF MEDICINE AND SURGERY.

### Puerperal Convulsions, from the Standpoint of Prevention.

BY JOHN N. UPSHUR, M. D., RICHMOND, VA.,
Professor Practice of Medicine in the Medical College of Virginia, etc.

[Read before the Richmond Academy of Medicine and Surgery, November 24, 1896.]

A firm conviction of the great responsibility resting upon us in the care of the puerperal woman, and the skillful guidance through the perils of this period to its happy consummation in a safe delivery, is the motive prompting to the discussion of this condition—so frightful in its manifestations and so dire often in its consequences.

I cannot emphasize too much the importance to the patient of an early engagement of her medical attendant, that she may be closely in touch with him, and that he, by *extraordinary diligence*, may be keenly alive to every circumstance which may be to her a source of peril. Giving to his patient advice as to personal care, of diet, exercise, rest, bowels and kidneys; making regular, often frequent, analyses of the urine to determine the presence of albumen or the lacking elimination of excrementitious solids. Often there is danger ahead when urinary analysis is negative in its results, and the nervous system is ripe for a dangerous explosion as soon as something occurs to excite the requisite reflex. I cite the following cases for sake of illustration:

Case 1.—Mrs. T., a woman of fine physique and robust health, came under my care in 1885. She would never permit me to see her until labor came on, though reported by her husband as being very dropsical. Frequent examination of her urine failed to show any albumen, though the amount of urine was scanty. Treatment, through the medium of her husband, was, of course, very unsatisfactory. When labor came on, I found her the most dropsical woman I ever saw at term. She was kept under chloroform until delivered with instruments, after a protracted labor. In four succeeding pregnancies she was never so dropsical again, but each period of pregnancy was filled with symptoms of threatened eclampsia. The patient was a hearty feeder, her urine was usually scanty, albumen apparent on analylis, but not marked.

In her third pregnancy she had an attack of vertigo, followed by a period of insensibility; she had always suffered with her head. Her treatment during each pregnancy consisted of low diet, abundant drinking of lithia water, and keeping bowels in a condition of mild diarrhœa. She has usually taken chloroform during the latter part of the second stage of labor, and has been safely delivered five times without a single convulsion, and is now doing well in her sixth pregnancy, having reached the end of the sixth month.

Case 2.—Mrs. W., of delicate build, in her second pregnancy, during the ninth month had agonizing headache, which failed to respond to remedies; saw flashes of light and other objects continually before her eyes. When labor came on, she was kept under chloroform until delivered; her skin was so dry as to feel parched. Repeated examination of urine failed to detect any albumen. Within an hour after delivery, she complained of not being able to see (she had had no undue amount of hemorrhage), and began to talk wildly and incoherently. Chloroform was given to control excitement, followed by full doses of potassium bromide and pilocarpine; skin acted freely, and all untoward symptoms subsided; urine showed large percentage of albumen. Convalescence uneventful until fourteenth day, when marked symptoms of septicæma developed, but she passed safely through a most dangerous illness. (See *Transactions State Medical Society of Virginia*, 1886.)

Case 3.—Mrs. H., had been carefully watched during second pregnancy, urine frequently analyzed, with negative results. About the time she reached the fifth and a half month, I was called to see her with a sharp attack of cholera morbus; a day or two of treatment was sufficient to restore her to health. She was discharged, with caution as to imprudence in diet, and every other respect. On the evening of next day, she went out to supper, eat very heartily of almonds and raisins. I was called early the following morning to see her in what her husband called a *trance.* When I reached her, she talked to me rationally, and manifested no symptoms of special gravity. At 4 p. m., she was seized with a violent puerperal cunvulsion, quickly followed by a second, and for a few moments I thought her dead. I bled her freely from the arm, labor was brought on, and after delivery she made an uneventful recovery—urine for the first time loaded with albumen.

Her next pregnancy progressed satisfactorily to the ninth month, when she complained of distressing head symptoms, urine scanty, but free from albumen; some disturbance of vision. She was freely purged with calomel gr. vj., croton oil gtt. j., with complete relief of head symptoms. The week following, head symptoms again returned, and the dose of calomel and croton oil was repeated, with as prompt relief as at first. Labor came on a few days after, and she was safely delivered without any complication; convalescence speedy and uneventful.

Case 4. Mrs. D., primipara, æt. 22, stout and plethoric. I was retained six weeks before confinement. Frequent analysis negative, except once a trace of albumen, until October 12th, when urine was found loaded with albumen, being almost solid on boiling. Was called at 10 p. m.; found she had had three convulsions, cervix rigid, dilated to size of a nickel. She was bled freely from the arm, manual dilatation persisted in for five hours; child turned and delivered; a serious convulsion during the labor, which looked as though it would be fatal; no untoward symptoms during convalescence. I found she had been complaining for a week before the labor with violent headache. I had not been consulted. At the same time she had been eating enormously.

Case 5.—Was called in consultation to see Mrs. H., primipara, æt. 22. Saw her at 6:30 p. m. She gave the history of an unusually comfortable pregnancy. Had engaged her medical attendant *two days* before; said she had passed sufficient urine. Awoke at 5 a. m. same morning with severe headache across vault of cranium, extending over occiput and down back of neck, and some abdominal pain, which was supposed to be colic, as her time was not up (280 days) till two weeks later. Had been abstemious in her diet during latter part of pregnancy, abstaining from eating any supper. Just before 3 o'clock p. m. she described a sensation in her head as if blood was trickling through it, and a few moments later a violent eclampsic seizure developed. Her medical attendant, when he saw her in the morning, had ordered $\frac{1}{8}$ gr. doses of sulph. morphia every two hours—she having taken in all about $\frac{3}{8}$ gr. So soon as he saw her after the convulsion, he bled her freely and administered chloroform, controlling the convulsion. When I saw her she was completely relaxed, os fully dilated and bag of waters filling the vagina—ruptured accidentally in my examination. Labor progressed satisfactorily and she was delivered of a live baby at 8:15 p. m.—skin hot and dry, pulse soft, feeble and 120 per minute—profoundly unconscious. Another convulsion at 9 p. m. No secretion of urine since early morning; an enema of bromid. potass., sulph. morph. and camphor was administered, and she passed into a quiet, natural sleep, having a good night with the exception of slight restlessness. She had also a hypodermic of $\frac{1}{20}$ gr. sulph. strychnia. On next day she had three more convulsions but of diminishing severity and longer interval; vision impaired, and she was unable to recognize her friends before the third day; periods of consciousness alternated with periods of delirum.

The baby had one convulsion on the day subsequent to its birth, but afterwards did well.

REMARKS.—It is not my purpose to discuss the classification or causes of eclampsia; this is sufficiently done in all modern text books. It is in the direction of such *care as will prevent the occurrence of convulsions* that I wish to consider the subject.

The cases cited as illustrative, emphasize—first, the necessity of early engagement, and subsequent close supervision. Case 1 shows how such care warded off trouble in five pregnancies. Case 4 points to threatened trouble indicated by the severe head symptoms with negative evidences from the urine, and with case 5 emphasizes this symptom and the hot, dry skin—relief coming when the skin and kidneys had their functions restored. Cases 3 and 4 point to the necessity of careful dieting, and the overloading of the stomach as an exciting cause—in the one case, no albumen, and in the other albumen only twice discovered prior to the development of convulsions. Where there is excessive eating, a hypernutrition is the result in a system in which there is already a hyperplastic condition of the blood. The attempt on the part of the kidneys to eliminate increased effete matter begets kidney irritation—it fails in its function, and the appearance of albumen in the urine is the external manifestation of poisoning of the system by toxines, which, unless eliminated, result in an eclampsic explosion.

The patient's diet should be nutritious, digestible, not rich; regular exercise in the fresh air, and especial care of the bowels and kidneys.

The administration of saline cathartics should be frequent, making the mucous membrane of the bowels eliminative and derivative; an abundance of lithia water and other necessary diuretics, to stimulate the kidneys. If the patient shows evidences of anœmia full doses of the tinct. chloride of iron long continued will be of value.

The treatment of the case when convulsions occur, I believe, consists imperatively in free administration of chloroform and prompt bleeding, with active purgation if the bowels are constipated; nor should we forget that the welfare of the patient depends upon as *prompt* delivery as possible—by manual dilation of the womb and forceps or turning. When, as in case 5, the patient remains unconscious after delivery, with symptoms of depression, as evidenced by rapid, feeble pulse, a hypodermic of strychnia nitrate will do as much good by its action in sustaining the heart and diminishing cerebral congestion by its toning up effect on the vasco-motor nervous system, and at the same time sustaining the uterus in firm contraction.

I am convinced of the usefulness of morphia in conditions of rigid os and cervix in the first stage of labor—*but*, if there be symptoms of threatened eclampsia, *it is positively contraindicated, and should not be given* in the treatment of the convulsions; it arrests secretion in the skin and kidneys, and favors the retention of the effete materials in the system.

DISCUSSION.

Dr. Jacob Michaux said he was sorry he was not present to hear the whole of the interesting and instructive paper, but he wished to speak of several things of which no mention had been made:

First, as to the causation of puerperal convulsions. Conclusions as to the retention of urea in the blood were drawn from the experiment in which one hundred grains of that substance were injected into an animal without causing convulsions. He would say that, considering the amount of urea voided, the quantity was by far not large enough to produce the symptoms. A second theory of causation is the retention of other excreta, which is doubtless true, but he believed urea played an important part.

Regarding the morphine treatment, he was in thorough accord with Dr. Upshur. At one time it was thought proper, but considering its effect on the excretory organs, it could be immediately seen that the indications were thwarted.

A subject in which there was a great deal of misconception was, when convulsions were threatening, the custom was to use diuretics. The practice is wrong, for this reason: The uterus presses not necessarily on the renal, but on other abdominal vessels, the pressure being projected through intervening ones to those of the kidneys, which become congested, and albuminuria results. Using diuretics, the kidneys are further congested. Therefore, it is highly important to look to the bowels as the chief means for getting rid of the poisonous matter. He was confident that he had seen impending convulsions thwarted by the use of salines. He coincided, also, with Dr. Upshur in the use of the lancet and chloroform; but employing the former, care should be had in anæmic cases. He had had good results, too, from veratrum viride in conjunction with chloroform, as likewise from chloral and the bromides.

Dr. H. Stuart MacLean stated that Flint records an experiment in which a pound of urea was injected without production of convulsions, but the injection of urine produced them.

Concerning treatment, he said that during his service at the Brooklyn hospital, of seven cases of puerperal eclampsia, three in which veratrum viride had been administered, died, while four, not treated thus, recovered. In the latter, hot packs and bleeding were the measures employed. Where bleeding was done antepartum, high injections of saline solution, equal to the amount of blood drawn, and lavage of the stomach, were employed.

Dr. C. R. Robbins described a case in which there were all the premonitory symptoms of eclampsia. Primipara, eight and a quarter months' pregnancy, urine albuminous, frontal headache, epigastric pain, œdema, spots before the eyes, etc. The treatment consisted of an absolute milk diet, lithia water and nitroglycerin. The patient had no convulsions, and delivery was successful.

Dr. W. W. Parker concurred almost *in toto* with Dr. Upshur, but had more faith in veratrum viride. He descriqed the following cases:

Case 1, was in the second month of pregnancy, and when seen before the attack, there was no sign of trouble. Suddenly, there was violent headache, and in a half hour, convulsions. She died in twenty-four hours without recovering consciousness. He thought her death was due to apoplexy.

Case 2. Eclampsia occurred in the third month of pregnancy! The urine was loaded with albumin. The convulsions were treated, and while they never returned, two weeks after the attack, there were headaches, blindness, etc., and the albumin had increased until the urine was almost solid. Immediate production of labor was insisted upon. He thought the patient could not possibly have survived had it not been done.

Dr. John F. Winn asked if it was Dr. Upshur's custom to give pilocarpine, and if he had ever used the hot pack?

Dr. Upshur replied that the administration of pilocarpine depended on circumstances. As to the hot pack, he never used it, as it was almost impossible in private practice. It was not because of lack of faith that he did not use it.

Dr. Winn continuing, said there was one phase of treatment that he had not heard touched upon, and that was early emptying of the uterus. When convulsions were present, there was no difference of opinion. When imminent and not yielding to ordinary treatment, they would end in premature labor. Therefore, following nature's plan, would it not be well to induce it, thus putting a stop to the convulsions, and relieving the woman of a liability to further attacks?

He described a fatal case in which convulsions did not occur until an hour after labor. The hot pack, venesection, chloral and bromide injections were all employed. He thought the induction of premature labor was always called for, both in the interest of mother and child, in every case whose symptoms were not relieved by dietetic and medicinal measures.

Dr. W. T. Oppenhimer thought the causation had a great deal of bearing on the case. Thus, short women are more susceptible than others. Always there is some dyspepsia of the small intestine giving rise to fermentation, which, with the gravid uterus, presses on the blood vessels of the kidneys, producing atrophy. These cases he treated by anti-ferments, digestives, etc. Salines were employed also. Women not disposed to alimentary troubles, are seldom affected. He advocated bleeding and the use of alkalies.

Dr. Arthur Jordan, apropos of the injection of urea, remarked that the experiments had been made with rabbits, and that it was supposed, too, that the urea passed off too rapidly. The renal arteries were ligatured, but no satisfactory results were obtained.

He thought the trouble was due to hyper-sensitiveness of the nervous system, no matter how it may be brought about. It behooves us, therefore, to watch anything that has a deleterious effect. The treatment went to favor his statement—viz: chloroform, chloral, bromides, which kept in subjection the nervous system. A remedy he saw used with happy results, was morphine in one-half grain doses until a grain and a half to two grains were given.

Dr. Edward McGuire reported the following case: The patient was somewhat anæmic, and in the eighth month of pregnancy. She progressed well until July, when, without known cause, there was paresis of the corner of the mouth, uvula and tongue. The diagnosis was embolism of the vertebral artery. Since that time there had been excessive nervousness, the least movement of the child bringing on a nervous attack. In September, while out of town, she had a convulsion, and the physician called in gave chloral. He saw her three hours later. There was no albumen in the urine. A short time ago, two months after the first, she had a second attack in the night. She had complained to her husband of pain in the face, and a feeling of impending convulsions. He saw her when the attack had passed.

As she suffered from photophobia, the light was low. Entering the room, he turned on the gas, and she immediately went into another convulsion. He doesn't know what form it was. Since this, she has had no other attack. The amount of urine was normal, and there was no albumen. He hesitated to induce labor. She never had a convulsion before pregnancy.

Dr. Hugh M. Taylor said that venesection and transfusion appealed more to him than any other treatment. We want no better evidence of the uncertainty of treatment than its wide range. He reported the following case in advocacy of Dr. Winn's early induction of labor. The patient was short and fat. In the morning there was one pain, and the bag of waters broke. In the early night, she was very nervous, but recovered, and labor went on slowly, there being progression and recession. Suddenly there was a terrific convulsion. Chloroform was administered, and she laid in a stupor so long that it was hard to tell when the stertor of the convulsions stopped, and that of chloroform began. The uterus was emptied, and the patient recovered consciousness, and is now doing well. He did not mean to say that this sustained *in toto* the treatment. Albumin was never found.

Dr. W. S. Beazley described the following case, a II-para. On reaching it, he found labor far advanced, with no untoward symptoms, and everything went on normally. He left the house, but in two hours, having been sent for, he found the patient in a hard convulsion, which passed off in a few minutes. She did not regain consciousness, and, in a half hour, went into a second attack. Enemata of bromide of potassium and chloral were given, and chloroform administered, but to no avail, as she grew worse and died. There were no convulsions with the first labor.

Dr. Upshur, in conclusion, remarked that he wished to say that Dr. Oppenheimer had gotten the cart before the horse. The digestive symptoms of which he spoke, and the consequent accumulation of gas, were due to an attempt on the part of the stomach and bowels to relieve the system by vicariously eliminating effete matters which should go off by the kidneys; the irritation thus set up *caused* the indigestion and consequent flatus. The frequent vomiting, the renal asthma, etc., which we see in several forms of Bright's disease, is illustrative, the mucous lining of the stomach and bowels, and bronchioles, seeking to help the kidney. The pregnant woman's blood is in a condition of increased plasticity, there is over-nutrition and over-stimulation of the kidney, it thus fails in its eliminative function, and the presence of albumin in the urine is the external manifestation of this condition; toxic matters remain in the blood, poisoning the nervous system, and find expression in a convulsive explosion when the proper reflex comes, from an overloaded stomach, or the pains of labor.

